# On the Roles of Protein Intrinsic Disorder in the Origin of Life and Evolution

**DOI:** 10.3390/life14101307

**Published:** 2024-10-15

**Authors:** Vladimir N. Uversky

**Affiliations:** Department of Molecular Medicine and USF Health Byrd Alzheimer’s Research Institute, Morsani College of Medicine, University of South Florida, Tampa, FL 33612, USA; vuversky@usf.edu; Tel.: +1-813-974-5816; Fax: 1-813-974-7357

**Keywords:** intrinsically disordered proteins, protein–protein interactions, post-translational modifications, alternative splicing, structural heterogeneity, multifunctionality, membraneless organelles, liquid–liquid phase separation, origin of life, evolution

## Abstract

Obviously, the discussion of different factors that could have contributed to the origin of life and evolution is clear speculation, since there is no way of checking the validity of most of the related hypotheses in practice, as the corresponding events not only already happened, but took place in a very distant past. However, there are a few undisputable facts that are present at the moment, such as the existence of a wide variety of living forms and the abundant presence of intrinsically disordered proteins (IDPs) or hybrid proteins containing ordered domains and intrinsically disordered regions (IDRs) in all living forms. Since it seems that the currently existing living forms originated from a common ancestor, their variety is a result of evolution. Therefore, one could ask a logical question of what role(s) the structureless and highly dynamic but vastly abundant and multifunctional IDPs/IDRs might have in evolution. This study represents an attempt to consider various ideas pertaining to the potential roles of protein intrinsic disorder in the origin of life and evolution.

## 1. Introduction: Who Are You, Mr. IDP?

For most of its existence, protein science was ruled by the famous “lock-and-key” model proposed in 1894 by the German chemist Hermann Emil Louis Fischer (1852–1919) to describe the molecular mechanisms of enzymatic activity [1]. Here, the unique complementarity of the rigid structures of a substrate and an enzyme was suggested to define the efficiency of catalysis. Therefore, the specific functionality of a given protein was believed to be predetermined by the precise spatial positioning of its amino acid side chains and prosthetic groups, which, in its turn, was predestinated through a defined 3D structure of this protein (the so-called structure–function paradigm). Despite its numerous limitations, this structure–function paradigm, assuming that protein functionality is directly linked to its unique rigid 3D structure, acted as a ‘Big Bang’ that gave rise to the universe of modern protein science [2,3], a universe where ordered proteins with well-defined structures conduct well-defined functions in a “unique sequence—unique structure—unique function” manner.

However, even the most structured proteins, instead of being rigid crystal-like entities, represent dynamic systems with different degrees of conformational flexibility [3]. In fact, the 3D structures of ordered proteins determined by X-ray crystallography and many other ensemble-based techniques represent averaged pictures [4]. This is because proteins constantly undergo structural rearrangements originating from the fact that the conformational forces stabilizing the protein structure are weak and can be broken even at ambient temperatures due to thermal fluctuations [3,5], providing protein groups involved in such interactions with the ability to form new weak interactions with comparable energy [5]. Therefore, ordered proteins exist as dynamic ensembles of interchanging conformations, where structural rearrangements, being of relatively small scale, happen relatively fast (they occur typically in a time scale that is faster than the time required for structure determination by X-ray crystallography and many other physical techniques) [4].

It was also pointed out that not all structures deposited in the Protein Data Bank (PDB) [6] are defined throughout their entire protein lengths but instead contain regions with missing electron densities (i.e., portions of protein sequences missing from the determined structures) [7,8]. These regions of missing electron densities, being flexible or disordered in nature, are incapable of coherent scattering of X-rays. They are very common in the PDB, as less than 30% of PDB protein structures do not have them [9]. In addition to ordered proteins possessing different degrees of conformational flexibility and ordered proteins containing malleable/disordered regions of varying lengths, many biologically active proteins are characterized by a complete or almost complete lack of ordered structure under physiological conditions and exist as highly dynamic and heterogeneous conformational ensembles [5,10,11,12,13,14,15]. These IDPs and hybrid proteins containing ordered domains and various IDRs [16] are characterized by remarkable conformational heterogeneity and constitute a significant part of the protein kingdom [17,18,19,20].

Since IDPs/IDRs cannot spontaneously fold under the “physiological” conditions promoting folding of ordered proteins/domains, it was not surprising to find that the universe of protein amino acid sequences can be divided into at least two very different categories: sequences that naturally fold into ordered proteins or domains, and sequences that yield IDPs/IDRs [3,21]. Furthermore, the removal of the restrictions posed by the need to spontaneously fold into an ordered structure to become functional dramatically increased the sequence space available to IDPs/IDRs in comparison with the sequence space available to foldable proteins and domains [3,22]. Therefore, the amino acid sequences of structureless and ordered proteins are dramatically different [10,12,13,23,24,25]. For example, IDPs with extended disorder (so-called native coils and native pre-molten globules) were shown to be characterized by a low content of hydrophobic residues combined with a high content of similarly charged residues [12]. At the more grained level, the IDPs/IDRs were documented to be significantly depleted in the so-called order-promoting amino acids (Cys, Trp, Tyr, Ile, Phe, Val, Leu, His, Thr, and Asn) and enriched in the disorder-promoting Ala, Gly, Asp, Met, Lys, Arg, Ser, Gln, Pro, and Glu residues [10,13,24,25,26,27,28]. These and other disorder-specific peculiarities of the amino acid sequences were used to design numerous computational tools for the reliable prediction of intrinsic disorder in proteins [10,13,17,29,30,31,32,33,34,35]. The use of those tools has opened a way to evaluate the natural prevalence of protein disorder, revealing that many proteins are expected to contain long IDRs and that the eukaryotic proteomes have a higher fraction of intrinsic disorder than prokaryotic proteomes [17,18,20,36,37,38,39,40]. It was also pointed out that these differences in disorder distribution within the protein universe can be understood by taking into account the facts that IDPs/IDRs have evolved to have specific functions, being commonly involved in regulation, recognition, and signaling (see below), and that the eukaryotes and especially in multicellular eukaryotic organisms possess complex and well-developed regulation networks that might rely on the capability of IDPs/IDRs to perform the necessary regulatory functions [5,19,41,42]. In fact, being commonly involved in the recognition, regulation, and control of various signaling pathways [41,42,43], IDPs/IDRs have a unique functional arsenal that is parallel and complementary to the catalytic and transport functions of ordered proteins [24,44,45,46].

## 2. Roles of Intrinsic Disorder in the Origin of Life

### 2.1. Prebiotic Life on Earth: Intrinsic Disorder of Extraterrestrial Peptides

Since glycine, among other molecules, was detected in comets, meteorites (see [47,48,49]), and the interstellar medium [50], and since oligoglycine can be synthesized on the surface of cold solid particles (cosmic dust) [51], one can assume that extraterrestrial biomolecules contributed to the origin of life on Earth [52]. In fact, CO (carbon monoxide), carbon (C), and NH_3_ (ammonia), which are the three most abundant species in the star-forming interstellar medium, were shown to condensate on the surface of cold dust grains and form isomeric glycine monomers in a barrierless manner; these can then polymerize to produce homo-polymeric peptides of different lengths even at low temperatures under astrophysically relevant conditions in the absence of irradiation or water [51]. Therefore, polypeptides of significant lengths, and not just elementary amino acids such as glycine, may be synthesized in rocky planets in the habitable zone and may have served as an important element when life as we know it originated ~4 billion years ago (see [53]).

It is unclear whether more complex heteropeptides can be synthesized via the mechanism proposed for extraterrestrial polyglycine synthesis [51]. However, meteorites (particularly carbonaceous chondrites) were shown to contain various amino acids. For example, 52 different amino acids were found in the Murchison meteorite, among which 33 were unknown in natural materials, while eight were amino acids found in terrestrial proteins [54]. Furthermore, a 4641 Da amino acid polymer predominantly containing glycine and some hydroxy-glycine and alanine [55] and the 2320 Da meteoritic protein hemolithin containing two glycine strands, each 16 residues long, terminated by iron atoms and holding additional oxygen and lithium atoms [56] were found in the carbonaceous chondrite CV3 meteorites Acfer 086 and Allende.

Importantly, isomeric polyglycine-based peptides similar to ones of extraterrestrial origin were strongly predicted to be intrinsically disordered [52]. Therefore, homopolypeptides that can be synthesized extraterrestrially from glycine via the pathway proposed by Krasnokutski et al. [51] or by some other yet unknown mechanisms cannot be ordered. Obviously, this is not a big surprise as the glycine included in such polypeptides, besides being the simplest amino acid, is considered a disorder-promoting residue. It was also emphasized that such disordered polypeptides of extraterrestrial origin could have been present for a long time due to the absence of proteases in the abiotic environment of the primitive Earth [52]. Of course, a peptide bond can be decomposed via uncatalyzed hydrolysis involving direct attack of the peptide bond by water. However, the half-time of such uncatalyzed hydrolysis is expected to be as long as 600 years [57]. Furthermore, since the atmosphere of the primordial Earth was reducing and had no molecular oxygen or other reactive oxides, the primordial ocean did not contain molecular oxygen or other reactive oxides, which further slowed down the rate of spontaneous hydrolysis of the primordial peptides [52]. To conclude this section, it is tempting to hypothesize that extraterrestrial IDPs might have contributed to the prebiotic origin of life on Earth [52]. A more detailed description of this important concept will be provided in the subsequent sections.

### 2.2. Prebiotic Life on Earth: Intrinsic Disorder of Primordial Proteins

The complex 3D structures of modern ordered proteins represent the result of lengthy molecular evolution. What then can one say about the structures of primordial proteins? It is clear that the chances of the first polypeptides that appeared in the primordial soup of the primitive Earth to have unique 3D structures are negligibly slim. Instead, with a very high probability, such polypeptides were intrinsically disordered. We can find indirect clues supporting the validity of this hypothesis when looking at some known facts. Although the Earth formed about 4.5 billion years ago and became cool enough to potentially spawn life around 4.2 billion years ago, the first fossils are dated 3.85 billion years, raising the question of what was happening in those years in between. At the beginning of the 20th century, Alexander I. Oparin (1894–1980) [58] and John Burdon Sanderson Haldane (1892–1964) [59] proposed a model that constitutes a cornerstone of the theory of molecular evolution according to which some organic molecules could have been synthesized spontaneously from the gases of the primitive Earth’s atmosphere. Such abiotic production of organic molecules would require a reducing atmosphere and ample supply of energy in the form of lightning and/or ultraviolet light. The validity of this idea was demonstrated thirty years later when Stanley Lloyd Miller (1930–2007) and Harold Clayton Urey (1893–1981) conducted elegant experiments deservedly known now as the Miller–Urey experiments. These experiments showed that placing non-organic compounds such as water vapor, hydrogen, methane, and ammonia, which were believed to represent the major components of the atmosphere of the primordial Earth, into a closed system and running a continuous electric current through the system to simulate lightning storms, believed to be common on the early Earth, resulted in the appearance of various organic molecules, including some amino acids [60,61]. Importantly, only about half of the modern amino acids were synthesized in these Miller–Urey experiments [60,61], suggesting that the first proteins on Earth may have contained only a few amino acids.

**Figure 1:**
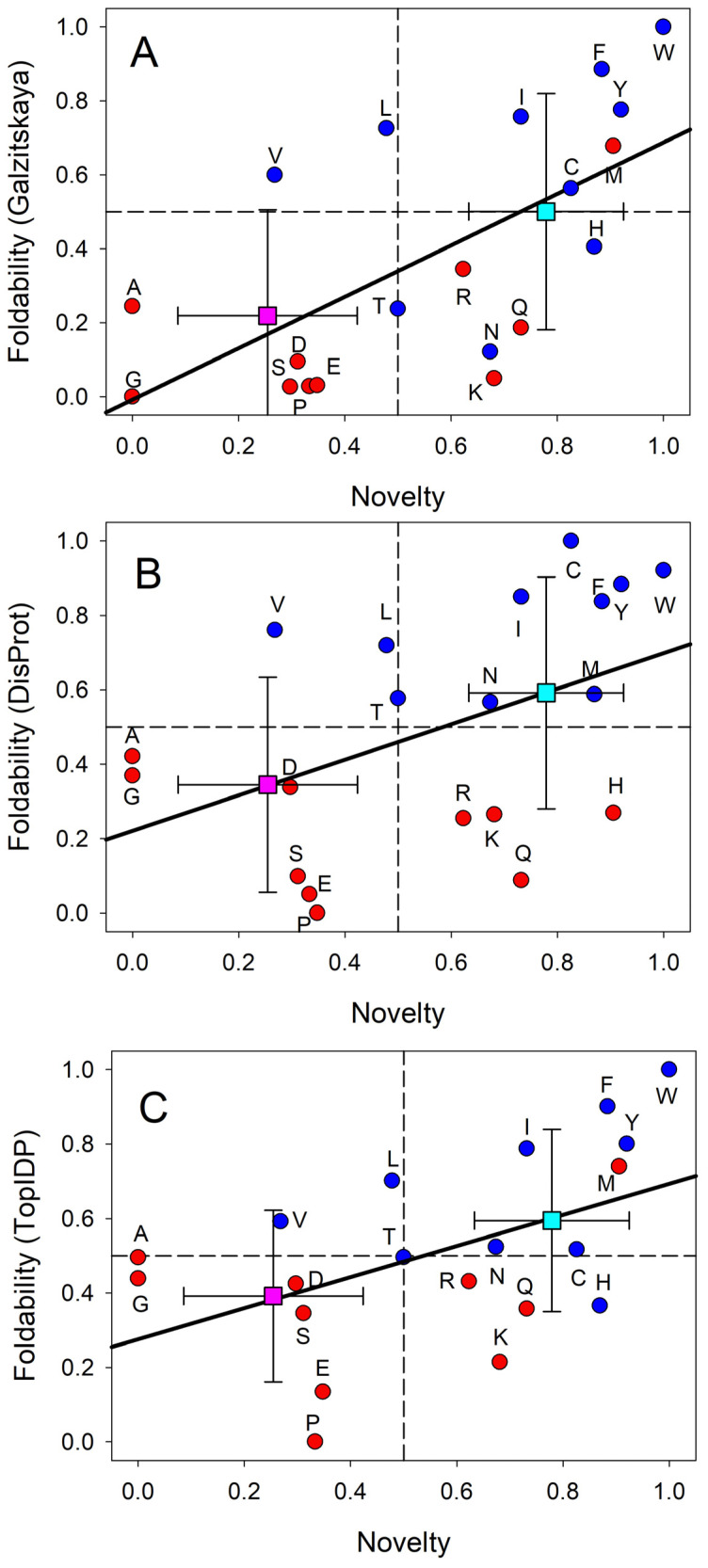
Correlations between three foldability scales (the scale based on the average number of contacts per residue in the ordered proteins (Galzitskaya) [67] (**A**), the DisProt-based scale [66] (**B**), and the Top-IDP scale [23] (**C**)) and amino acid novelty scale proposed by Trifonov [65]. Red and blue symbols correspond to disorder- and order-promoting residues as defined by the DisProt-based scale. Pink and cyan squares with error bars show averaged values.

In line with these considerations, the biosynthetic theory of the genetic code evolution suggests that the genetic code evolved from a simpler form encoding fewer amino acids [62], likely in parallel with the invention of biosynthetic pathways for new and chemically more complex amino acids [63]. Peculiarities of the redundancy of the standard genetic code, where 20 amino acids are encoded by 64 codons, provide some support to the validity of this hypothesis. Here, despite the fact that the redundant codons encoding one amino acid may differ in any of their three positions, only the third position of some of such codons may be fourfold degenerate, i.e., represents a position where all possible nucleotide changes are synonymous as they do not change the amino acid. If these peculiarities of the modern genetic code reflect its evolution, then it is likely that a doublet code preceded the triplet code, indicating that the third position was not used at all in the early genetic code. This means that this early code used 4 × 4 = 16 codons, thereby encoding 16 or fewer amino acids, if a termination codon is taken into account [64], indicating that evolutionarily old and new amino acids can be potentially discriminated. These and many other observations were used by Edward N. Trifonov to propose the following consensus order of the appearance of the 20 amino acids on the evolutionary scene: G/A, V/D, P, S, E/L, T, R, N, K, Q, I, C, H, F, M, Y, and W [65]. Let us look at this scale from the viewpoint of protein intrinsic disorder, where residues can be arranged based on their order-promoting (or foldability) potential [10,13,24,25,26,27,28]. In fact, there are three scales that can provide a ranking of the tendencies of amino acid residues to promote order or disorder: these are the Top-IDP scale (W, F, Y, I, M, L, V, N, C, T, A, G, R, D, H, Q, K, S, E, and P) [23], the DisProt-based scale (C, W, Y, I, F, V, L, H, T, N, A, G, D, M, K, R, S, Q, E, and P) [66], and the scale based on the average number of contacts per residue in the ordered proteins (W, F, V, I, L, M, V, C, H, R, T, Q, N, S, K, E, D, A, P, and G) [67]. Figure 1 represents a comparison of these scales with the amino acid novelty scale proposed by Trifonov and shows that typically, older residues (e.g., G, D, E, P, and S) have a strong tendency to be disorder-promoting, whereas many newer amino acids (e.g., C, W, Y, and F) tend to be order-promoting.

**Figure 2:**
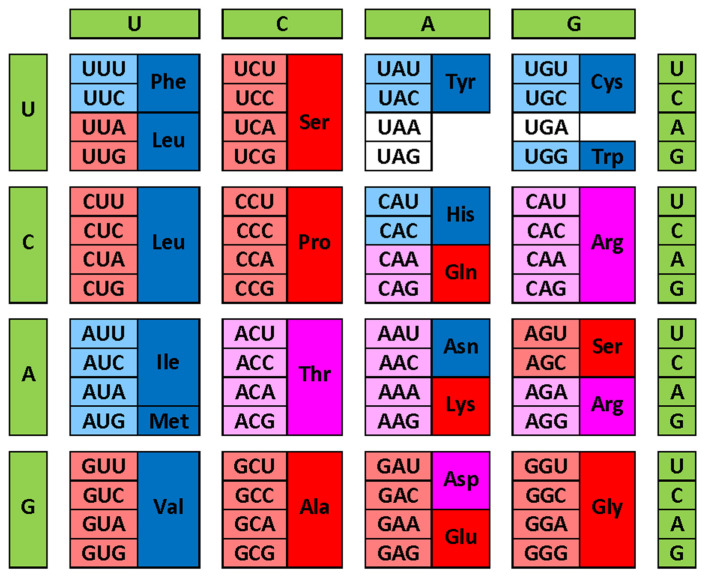
Modern genetic code with information on the early and late codons (shown by light red and light blue colors, respectively) and disorder- and order-promoting residues (shown by red and blue colors, respectively). Codons with intermediate ages (i.e., those located between early and late codons) are shown by the light pink color, whereas disorder-neutral residues are shown by the pink color. Adapted with permission from Ref. [3]. Copyright © 2013. The Protein Society.

Figure 2 provides another view of these correlations by showing modern genetic code complemented with information on the early and late codons (shown by light red and light blue colors, respectively) and on corresponding disorder- and order-promoting residues as evaluated based on the DisProt scale (shown by red and blue colors, respectively). Codons with intermediate age and disorder-neutral residues are shown by light pink and pink colors, respectively. This presentation emphasizes that there is relatively good agreement between the “age” of the residue and its disorder-promoting capacity, with early residues being mostly disorder-promoting and the majority of late residues being mostly order-promoting. This conclusion follows from the abundance of matching colors (light red–red, light blue–blue, and light pink–pink). There are only two noticeable exceptions to this rule, V and L, which are early but order-promoting residues.

**Figure 3:**
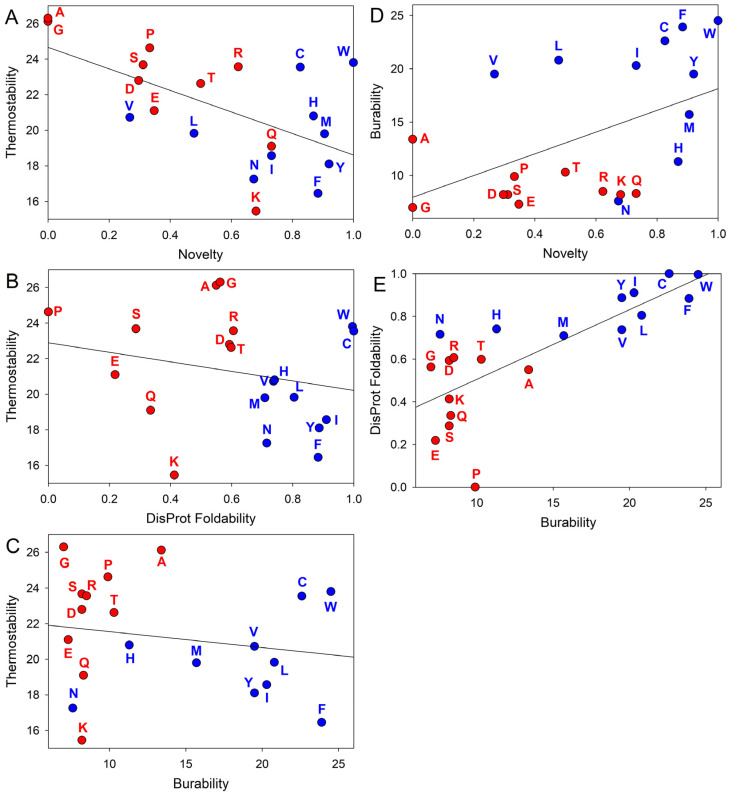
Correlations between thermostability of the codons (measured as melting enthalpies (kcal/M) of the dinucleotide stacks corresponding to the first and second codon positions [68]) and amino acid novelty of corresponding residue (**A**), thermostability of codons and DisProt foldability of corresponding residues (**B**), and thermostability of codons and buriability of corresponding residues (**C**), buriability of amino acids and their novelty, (**D**), and DisProt foldability and buriability (**E**). Red and blue symbols correspond to disorder- and order-promoting residues as defined by the DisProt-based scale.

There are also other facts that can provide further support for this idea. Since in the early stages of evolution, the primordial Earth was likely hotter than in the current day, more stable codon–anticodon interactions (in the absence of additional stabilizing interactions) were more favorable under these early conditions with presumably higher temperatures [65]. Therefore, the thermostability of the codons (measured as melting enthalpies (kcal/M) of the dinucleotide stacks corresponding to the first and second codon positions [68]) should have at least some correlation with the amino acid novelty scale. Figure 3A shows that such correlation is indeed observed, as early amino acids are typically encoded by more thermostable codons. Furthermore, Figure 3B shows that there is also an inverse correlation between codon thermostability and the disorder-promoting capability of amino acids, with disorder-promoting residues being encoded by more thermostable codons. One can also add another angle here and bring into consideration residue buriability, which provides a quantitative measure of the driving force for the burial of an amino acid residue in proteins and thereby contributes to the conformational stability of ordered proteins [69]. Figure 3C shows that codon thermostability is inversely correlated with the buriability of the residues encoded by these codons, whereas Figure 3D illustrates the presence of a correlation between the buriability and novelty of residues, where old residues are expected to be less buriable whereas high buriability is characteristic of new residues. Finally, Figure 3E shows that the disorder-promoting residues are less buriable than the order-promoting residues.

Taken together, these observations indicate that the primordial polypeptides were intrinsically disordered, as evolutionarily old amino acids, encoded by more thermostable codons, were less buriable and mostly disorder-promoting. Although it is rather unlikely that these disordered primordial polypeptides possessed high catalytic activity [70], undoubtedly they played important roles in the origin of life and were crucial players in early evolution as well. In fact, as per the RNA world theory, enzymatic activity evolution involves the transfer of catalytic power from catalytic RNAs (known as ribozymes, with an exceptional illustrative example being given by a ribosome, which is an RNA enzyme actually catalyzing the formation of peptide bonds during protein translation, and which is defined as “a creature with a hundred of waggly tails” since its stability is supported by numerous ribosomal proteins, most of which are disordered in the unbound state and fold upon binding to ribosomes [71]) to ribonucleoproteins (RNPs) and then to proteins [72]. Based on these premises, in an organism that was the first to invent protein synthesis, the first proteins would be IDPs with some nonspecific RNA chaperone activities rather than specific catalysts [70,73]. However, in the RNA world, where misfolding-prone RNA [74,75] was used for both information storage and catalysis [76], the presence of such disordered RNA chaperones would be highly beneficial to their carriers, providing them a significant selective advantage. Furthermore, the transfer of enzymatic activity from RNAs (ribozymes) to proteins was a logical evolutionary step determined by the higher stability of protein structures than RNA structures and by the dramatic increase in the variability of physicochemical properties of amino acids in comparison with those of nucleotides. Since a stable structure represents an important prerequisite for the proper spatial arrangement of catalytic residues, which is needed for efficient catalysis [77], transferring catalytic activities to proteins generated strong evolutionary pressure toward proteins with well-folded structures.

## 3. Roles of Intrinsic Disorder in Evolution

### 3.1. Wavy Evolution of Intrinsic Disorder: Back to the Future or Blast from the Past

**Figure 4:**
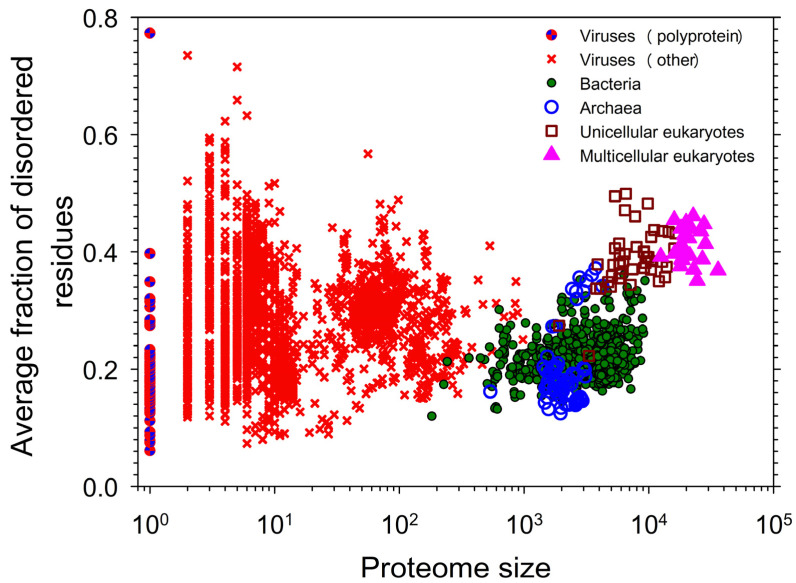
Correlation between intrinsic disorder content and proteome size of 3484 species of viruses, archaea, bacteria, and eukaryotes. Each symbol indicates a species. There are six groups of species: viruses expressing one polyprotein precursor (small red circles filled with blue), other viruses (small red circles), bacteria (small green circles), archaea (blue circles), unicellular eukaryotes (brown squares), and multicellular eukaryotes (pink triangles). Each viral polyprotein was analyzed as a single polypeptide chain, without parsing it into the individual proteins before predictions. The proteome size is the number of proteins in the proteome of that species and is shown as the log base. The average fraction of disordered residues is calculated by averaging the fraction of disordered residues of each sequence over all sequences of that species. Disorder prediction is evaluated by PONDR-VSL2B. Adapted with permission from Ref. [3]. Copyright © 2013 The Protein Society.

Figure 4 represents a snapshot of the distribution of intrinsic disorder in the modern proteomes [20] and illustrates the well-known fact that IDPs/IDRs are more prevalent in eukaryotes than in less complex organisms [17,18,36,37,38,39,40]. As already pointed out, this plot, representing the dependence of the fraction of disordered residues on the proteome size, has a well-defined gap between the prokaryotes and eukaryotes, as the majority of the prokaryotic species have 27% or fewer disordered residues, whereas almost all eukaryotes are predicted to have 32% or more disordered residues [20]. This observation indicates the existence of a complex stepwise correlation between the increase in organism complexity and the increase in the amount of intrinsic disorder and suggests that the “origination” of intrinsic disorder was crucial for moving from the less complex prokaryotic to more complex eukaryotic cells, which contain many intricate innovations that seemingly arose all at once. Therefore, the sharp jump in the levels of proteome disorder parallels a morphological gap between the prokaryotic and eukaryotic cells, indicating that the increased usage of intrinsic disorder paralleled and likely was crucial to the increase in the morphological complexity of the cell [20].

**Figure 5:**
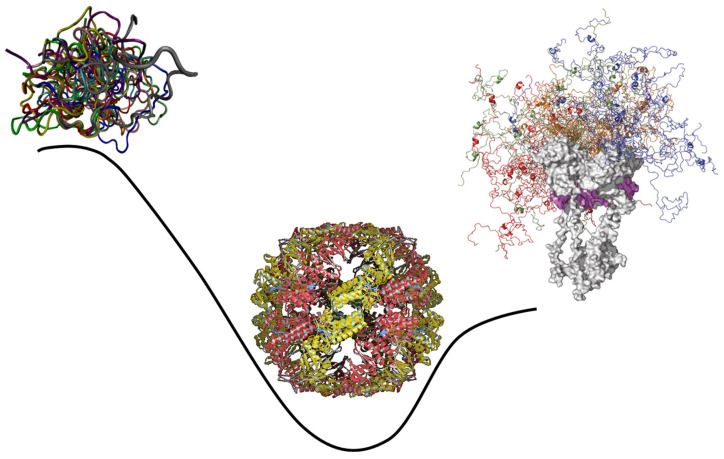
Wavy pattern of the global evolution of protein intrinsic disorder. The x-axis represents evolutionary time and the y-axis shows disorder content in proteins at a given evolutionary time point. Here, primordial proteins are expected to be mostly disordered (left-hand side of the plot), proteins in LUA likely are mostly structured (center of the plot), whereas many proteins in eukaryotes are either totally disordered or hybrids containing both ordered and disordered regions (right-hand side of the plot). Adapted with permission from Ref. [3]. Copyright © 2013 The Protein Society.

These observations clearly indicate that IDPs/IDRs, with their ability to control various signaling, recognition, and regulation pathways and networks, act as crucial life maintainers in eukaryotic organisms, especially multicellular eukaryotic organisms [5,19,41,42]. They also seem to suggest that the introduction of intrinsic disorder represents a relatively recent evolutionary “invention” that helped the move from prokaryotes to eukaryotes. However, as was discussed in the previous section, more likely than not, primordial proteins/polypeptides were intrinsically disordered. Therefore, the increased use of intrinsic disorder in eukaryotic organisms clearly represents a blast from the past and can be considered a “back to the future” event. This is illustrated by Figure 5 schematically showing that the pattern of the global evolution of intrinsic disorder is not straight, but wavy. Here, evolution starts with the highly disordered primordial proteins primarily acting as RNA chaperones. Since the competitive advantage of primitive cells was likely defined by the degree of their independence from the fluctuating environmental conditions linked to the ability to catalyze the production of all the constituents necessary for their independent existence, highly disordered RNA chaperones evolved into the ordered enzymes with well-folded unique 3D structures. At the subsequent evolutionary steps, protein intrinsic disorder was reinvented because IDRs/IDPs have specific features crucial for the regulation of complex processes. This prompted the development of more complex organisms from the last universal ancestor (i.e., the most recent organism from which all organisms now living on Earth descend [78,79]), eventually leading to the advent of the highly elaborated eukaryotic cells.

### 3.2. Intrinsic Disorder and LLPS: From Prebiotic Life to Origin of Cellular Life and Evolution

The aforementioned Miller–Urey experiments demonstrated that simple building blocks (including amino acids) required for the formation of complex macromolecules could form in environments seemingly mimicking early Earth [60,61]. These amino acids could have naturally assembled into polypeptide chains without the need for complex biological machinery. The principle possibility of such prebiotic peptide synthesis has been studied for decades, with researchers investigating different geological settings such as volcanic geothermal fields, hydrothermal fields, sea-floor sediments, and tidal flats [80,81,82,83,84,85] and also looking at the effects of minerals, salts, ions, and pH [80,81,82,83]. Under highly alkaline conditions, peptide synthesis was favored, and the 20-mer oligopeptides (Gly_20_—with no doubts, this was an IDP!) were synthesized [86]. However, such highly alkaline conditions could not support RNA synthesis due to the low stability of this biopolymer. Another attractive possibility was recently demonstrated in the experiments conducted by Yuki Sumie, Keiichiro Sato, Takeshi Kakegawa, and Yoshihiro Furukawa, who have shown that boric acid can catalyze polypeptide synthesis under neutral and acidic conditions, leading to the appearance of 39-residue-long glycine polypeptides (Gly_39_—IDP again!) [87]. These observations suggested that in the primordial Earth, polypeptides and proto-proteins could be spontaneously formed from the assembled amino acids in the coastal areas of ancient small continents and islands rich in boric acid [87]. Furthermore, it was indicated that “the same conditions would allow for the formation of RNAs and interactions of primordial proteins and RNAs that could be inherited by RNA-dependent protein synthesis during the evolution of life” [87]. These experiments provided important clues on how early chemistry could have evolved into self-replicating structures. Importantly, the phase separation of primitive macromolecules into liquid coacervates was proposed in the 1920s by Alexander I. Oparin as the first step in the origin of life [58,88].

Therefore, it is likely that primordial IDPs in general (and polyglycine in particular), liquid–liquid phase separation (LLPS, see below), and membraneless organelles (MLOs, see below) played crucial roles in prebiotic evolution. In fact, it was pointed out that polyglycine, with its ability to phase separate and form membraneless droplets and amyloid accretions, very likely contributed to the organization of the protocell domains, facilitation of the evolution of the genetic code, and the overall transition of the pre-life to the cellular life [89]. IDPs in the form of extraterrestrial polypeptides or the primordial IDPs abiotically synthesized on the early Earth could cause the emergence of self-organizing systems that evolved over time following natural selection [90,91,92]. Consistent with this hypothesis, a recent study by Matsuo and Kurihara [93] showed that under appropriate conditions, peptide generation and self-assembly occur concurrently and can give rise to a proliferating peptide-based droplet through liquid–liquid phase separation in water. Furthermore, it was observed that the droplets experienced steady growth–division cycle by periodic addition of monomers through autocatalytic self-reproduction [93]. It was also emphasized that LLPS “may represent a primordial mechanism for functional self-assembly of relatively unevolved molecular assemblies in the early stages of the evolution of life” [94].

LLPS-driven primordial coacervate formation did not wane during evolution. Instead, it seems that its fate is similar to that of IDPs. This is reflected in the fact that although different MLOs are found in the cells of all kingdoms of life, the variability of these biomolecular condensates is dramatically increased in eukaryotic cells, as most of the 100+ currently known MLOs/BCs are of eukaryotic origin [95]. A very important aspect related to the functionality of IDPs and IDRs is their crucial role in the regulation and control of LLPS, an important process associated with the biogenesis of various MLOs and biomolecular condensates (BCs) [94,96,97,98,99,100,101,102,103,104,105,106]. In fact, more than a hundred different MLOs/BCs can be found in the cytoplasm, nucleus, and mitochondria (and chloroplasts) of eukaryotic cells, as well as in the cytoplasm of bacteria and archaea, and, likely, in viruses [95], where they represent “an intricate solution of the cellular need to facilitate and regulate molecular interactions by physically isolating target molecules in specialized compartments in a reversible and controllable way” [102]. IDPs/IDRs are central constituents of all the MLOs investigated so far [98,101,102,107,108,109,110], as their structural plasticity and capability to be involved in multivalent, stochastic, weak, palpation-like interactions are crucial for LLPS, leading to the spontaneous separation of a homogeneous solution into two distinct immiscible liquids or “phases”: a dense phase and a dilute phase, both characterized by high water content and not separated by the membranes. As a result, MLOs always contain IDPs despite the fact that they differ from each other by the specific sets of their resident proteins [102]. It seems that the formation of MLOs/BCs often represents a way of the intracellular compartmentalization of IDPs/IDRs [101,102,108,111,112]. Being liquid in nature, MLOs are characterized by high levels of internal dynamics [94,96,113,114,115,116,117], thereby representing fluid disorder-based ensembles. Since MLOs can be formed on linear cellular structures such as chromatin and cytoskeleton, or in/on the membranes, or in the bulk of the nucleoplasm/cytoplasm/matrix/stroma, they are classified as 1D, 2D, or 3D assemblages that can influence each other, thus representing important pathway for intracellular communication and regulation [118].

It is clear that the protein intrinsic disorder, biological phase separation, and MLO phenomena are interlinked [102,106,107,118,119] since LLPS of specific IDPs is required for the formation of many (if not all) MLOs [98,102,111,120,121,122,123,124,125]. It was pointed out that this IDP/IDR-LLPS-MLO interconnection is redefining the organizational principles of living matter from a rather mechanistic model, where functions of proteins are determined by their rigid globular structures and where intracellular processes occur within the rigid membrane-encapsulated organelles, to a new model, where highly dynamic “biological soft matter” (IDPs and MLOs) positioned at the “edge of chaos” represents a critical foundation of life and defines complexity and evolution of the living things [107].

### 3.3. Intrinsic Disorder in Nucleic Acid-Binding Proteins

The textbook truism defines genetic programming as a classic molecular biology dogma, where genetic information flows from DNA to RNA to protein. However, it is clear now that this straightforward DNA → RNA → protein information flow, being an oversimplification, is mostly applicable to simple organisms. In fact, using it, one can understand how the *E. coli* genome works, as bacterial genomes mostly contain the information required for making proteins (typically, ~90% of bacterial genomes are responsible for protein coding). However, the eukaryotic genomes are immensely more complex, as reflected in the facts that genes of higher organisms represent complex mosaics of coding (exons) and non-coding sequences (introns that are removed from the messenger RNA during the process of splicing and can be extraordinarily large, accounting for the majority of the DNA sequence in human genes [126]), all of which are transcribed [127,128,129], with exons covering around 2.8% of the human genome [126]. Curiously, although most of the non-coding DNA in the eukaryotic proteome was considered non-functional (therefore termed “junk DNA” [130,131]), it was eventually shown that the vast majority (at least 80%) of the human and mouse genomes are in fact transcribed and have assigned biochemical functions [132,133]. The majority of the genome sequences conserved between humans and other mammals correspond to the non-coding intergenic and intronic regions, rather than in the protein-coding exons themselves, thereby indicating that these non-coding sequences have critical roles in development and cellular processes. Furthermore, the relative amount of non-coding sequences was shown to increase consistently with the organism’s complexity [133], indicating that bacterial genomes are mostly dedicated to making proteins, whereas eukaryotic genomes are mostly dedicated to the production of non-coding RNAs with various regulatory functions. Therefore, especially in complex organisms, RNA not only acts as a passive, mostly linear, messenger between DNA and protein but is actively involved in the regulation of genome organization and gene expression [134]. In doing that, RNA can fold into specific 3D structures that are complex and can be allosterically responsive, and which “can both recruit generic effector proteins and guide the resulting complexes sequence-specifically to other RNAs and DNA” [134].

Obviously, most of the regulatory RNA functions are conducted in close conjunction with the RNA-binding proteins (RBPs), which are intimately involved in the regulation of gene expression, post-transcriptional regulation, and protein synthesis, as well as governing the maturation and fate of their target RNA substrates [135,136]. Furthermore, RBPs establish a specific network complementing a network regulating gene activity and differently organizing RNA transcripts in different tissues. The global importance of RBPs is reflected in the fact that the human proteome contains at least 1542 such proteins [135,136], indicating that RBPs represent the third major protein group in human cells, in addition to soluble globular proteins and membrane proteins. Based on the comprehensive bioinformatics analysis of ~548,000 proteins forming nucleiomes (i.e., sets of nucleic acid-binding proteins) in 1121 species of Archaea, Bacteria, and Eukaryota, it was concluded that the entire nucleiome is enriched in intrinsic disorder, as evidenced by significantly increased intrinsic disorder content in DNA- and RNA-binding proteins relative to other proteins in corresponding proteomes [137]. This global analysis supported conclusions of earlier studies focused on specific families and classes of DNA- or RNA-binding proteins, with some of the illustrative examples of intrinsically disordered DNA- or RNA-binding proteins being histones [138], ribosomal proteins [71], transcription factors [139,140,141], and proteins involved in the biogenesis and action of yeast [142] and human spliceosomes [143]. Furthermore, focused bioinformatics analysis of the prevalence of intrinsic disorder in human RBP binding to six common RNA types—messenger RNA (mRNA), transfer RNA (tRNA), small nuclear RNA (snRNA), non-coding RNA (ncRNA), ribosomal RNA (rRNA), and internal ribosome RNA (irRNA)—revealed that although RNA-binding proteins are generally enriched in intrinsic disorder, the disorder propensity is unequally distributed across proteins that bind different RNA types [144]. In fact, although the mRNA-, rRNA-, and snRNA-binding proteins were predicted to be significantly enriched in disorder, the proteins that interact with ncRNA and irRNA were not enriched in disorder, and the tRNA-binding proteins were significantly depleted in disorder [144].

## 4. Intrinsic Disorder as a Means of Increasing Proteome Complexity

### 4.1. Alternative Splicing

Alternative splicing is an important process by which two or more mature mRNAs are produced from a single mRNA by the inclusion and omission of different segments [145,146], which therefore serves as an important mechanism for enhancing protein diversity in multicellular eukaryotes [147]. For example, the tissue specificity of many proteins is achieved via alternative splicing. The process is very common especially in higher eukaryotes, with between 35 and 60% of human genes yielding protein isoforms by means of alternatively spliced mRNAs [148,149,150]. It was hypothesized that alternative splicing affects the diversity of protein functions, such as protein–protein interactions, ligand binding, and enzymatic activity [151,152,153]. In multicellular organisms, such added protein diversity from alternative splicing is important for tissue-specific signaling and regulatory networks.

**Figure 6:**
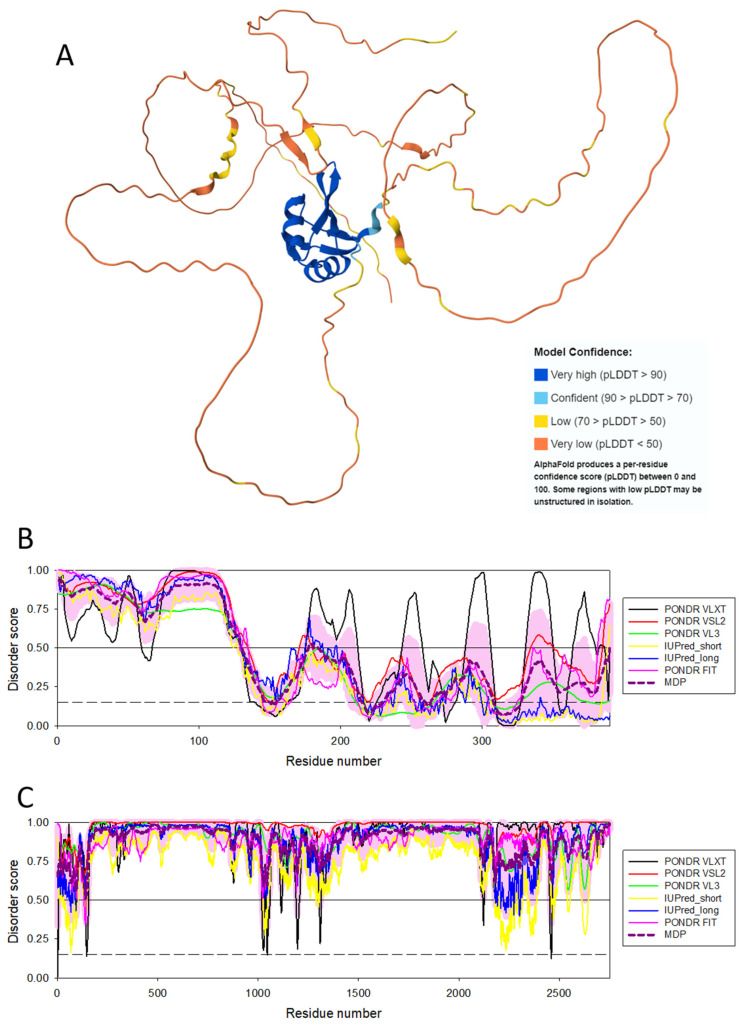
Intrinsic disorder in spliceosomal proteins. (**A**) The 3D structural model generated for one of the moon-lighting spliceosomal proteins RBFOX2 (UniProt ID: O43251) by AlphaFold [156]. The structure is colored according to the model confidence. (**B**) Per-residue intrinsic disorder profile of RBFOX2 generated by RIDAO [157]. (**C**) RIDAO-generated per-residue intrinsic disorder profile of spliceosomal protein SRRM2 (UniProt ID: Q9UQ35) involved in the biogenesis of nuclear speckles.

The aforementioned fact that the spliceosomal RBPs are enriched in intrinsic disorder [142,143] reflects the crucial importance of IDPs/IDRs in the splicing of the eukaryotic protein-encoding mRNAs, a process by which a spliceosome removes the non-coding regions (introns) from a pre-messenger RNA (pre-mRNA) transcript and joins the coding regions (exons) to create mature mRNA. Since during splicing, exons from the same gene can be joined in different combinations, leading to different, but related, mRNA transcripts, and since these alternatively spliced mRNAs can be translated into different proteins with distinct structures and functions, IDP-containing spliceosomes play crucial roles in the alternative splicing-driven increase in proteome complexity. Furthermore, because of their intrinsically disordered nature, many spliceosomal RBPs possess several unrelated functions, i.e., have the ability to moonlight, whereas some spliceosomal RBPs drive LLPS and the formation of various MLOs via interaction with RNA. To illustrate the disorder status of some of such spliceosomal intrinsically disordered RBPs, Figure 6 represents AlphaFold-generated 3D structural model for one of the moonlighting RBPs involved in the regulation of alternative splicing in nervous system, RNA binding protein fox-1 homolog 2 (RBFOX2; UniProt ID: O43251), which besides regulating the alternative splicing events by binding to 5′-UGCAUGU-3′ elements can also act as a negative regulator of the human estrogen receptor (ER) signaling and play a role in some ovarian cancers [154]. Figure 6B represents a per-residue intrinsic disorder profile generated by RIDAO and shows that human RBFOX2 is predicted to have high levels of intrinsic disorder, especially in its N-terminal region preceding the RNA recognition motif (RRM, residues 121–197). Figure 6C shows disorder profile for the spliceosomal RBP serine/arginine repetitive matrix protein 2 (SRRM2, UniProt ID: Q9UQ35) that serves as a component of the minor spliceosome and is thereby required for pre-mRNA splicing but is also involved in the biogenesis of nuclear speckles (NS), which are among the most prominent biomolecular condensates [155]. Figure 6C leaves no doubt that SRRM2 is an extremely disordered protein. Curiously, the region comprising residues 197–259, which is sufficient for RNA binding, is predicted to be mostly disordered as well.

Importantly, IDPs/IDRs are not only crucial for the control and execution of alternative splicing of precursor pre-mRNAs but also have a vital role in another side of this phenomenon, as protein regions affected by alternative splicing of pre-mRNAs are enriched in intrinsic disorder [158]. The fact that alternatively spliced segments of mRNAs mostly encode IDRs provides an important means for avoiding potential conformational catastrophes. This is because, in ordered proteins capable of spontaneous folding, most of the amino acid sequence contributes to the folding process and is involved in structural stability, as the specific sequence determines which interactions can form between amino acid residues, ultimately shaping the 3D structure of a protein. In other words, the information contained in a protein amino acid sequence determines its unique 3D structure and thereby acts as a specific protein folding code. Therefore, it is likely that the removal of a piece of an amino acid sequence of a foldable protein containing a part of the said folding code (e.g., as a result of alternative splicing of the corresponding mRNA) would distort the capability of a protein to spontaneously fold in the right structure, causing the aforementioned conformational catastrophe reflected in protein misfolding, aggregation, and associated issues. However, no conformational catastrophe is expected if the protein/region is intrinsically disordered, as the removal of a piece with “no structure” would have much of an effect on the remaining “no structure”. On the other hand, it was proposed that associating alternative splicing with protein disorder enables the time- and tissue-specific modulation of protein function [158]. Since IDRs are frequently utilized in protein-binding regions, having alternative splicing of pre-mRNA coupled to IDRs can define tissue-specific signaling and regulatory diversity [158]. Furthermore, since regulatory and signaling elements of IDPs/IDRs can be as short as just a few residues, and since functionally important segments can be located within the IDRs with a high density, the functionality of IDPs/IDRs can be completely rewired via alternative splicing [158]. Therefore, a linkage between alternative splicing and signaling via IDRs represents one of the possible molecular mechanisms that led to the origin of cell differentiation, which ultimately gave rise to multicellular organisms [158].

### 4.2. Post-Translational Modifications

In addition to the aforementioned alternative splicing, the complexity of a proteome relative to its encoding genome is known to be dramatically increased via various posttranslational modifications (PTMs) of proteins. These spontaneous or enzymatically catalyzed chemical changes of a polypeptide chain happen after DNA has been transcribed into RNA and translated into protein and can be reversible or irreversible. PTM-related increases in proteome complexity are determined by the capability of PTMs to extend the range of amino acid structures and physicochemical properties, thereby leading to the diversification of protein structures and functions [159]. It is emphasized that because of various PTMs, proteins might contain more than 140 physicochemically different residues despite the fact that 20 primary amino acids are typically encoded by DNA [159]. It was also indicated that there are as many as 300 physiologically relevant PTMs in higher eukaryotes [160]. Although all amino acid side chains can serve as PTM targets, most commonly, protein PTMs are found at side chains that can act as either strong (C, M, S, T, Y, K, H, R, D, and E) or weak (N and Q) nucleophiles, whereas the remaining residues (P, G, L, I, V, A, W, and F) are rarely involved in enzymatically catalyzed covalent modifications of their side chains [159]. Furthermore, since some commonly observed PTMs (e.g., phosphorylation and glycosylation) are readily reversed by the action of specific demodifying enzymes, the interplay between the corresponding modifying and demodifying enzymes provides an important means for rapid and economical control of protein function.

The overall importance of PTMs in various aspects of the cellular “life” of proteins is reflected in the fact that as much as 5% of the eukaryotic genomes are expected to encode PTM-related enzymes [160]. In fact, some PTMs are known to regulate the process of protein folding, whereas other PTMs control protein targeting to specific subcellular compartments and interaction with ligands or other proteins, and still other PTMs manage protein functional states affecting catalytic activity of enzymes or the signaling potential of proteins in various signal transduction pathways [161,162]. It is estimated that phosphorylation/dephosphorylation cycles originating from carefully regulated protein kinase and phosphatase activities control the functions of one-third of eukaryotic proteins [163]. Not surprisingly, eukaryotic protein kinases constitute one of the largest protein families, where yeast, mouse, and human kinomes include 119, 540, and ~520 kinases, the human genome contains more than 150 genes encoding phosphatases, whereas there are 1019 kinase- and 300 phosphatase-coding genes in *Arabidopsis thaliana* [163]. The functionality of some proteins is controlled by multiple different PTMs that can act individually or synergistically to fine-tune molecular interactions and modulate overall protein activity and stability [164]. An illustrative example of well-known multi-PTM proteins is given by a family of nuclear IDPs, histones, that are known to undergo acetylation, ADP-ribosylation, methylation, phosphorylation, SUMOylation, and ubiquitylation at different stages of their function [138]. Although for a long time the N-terminal tails of the core histones containing an extraordinary number of different PTMs were known to play important roles in the nucleosome dynamics and related gene expression and transcription [165], over 30 PTMs have been reported in the core domains of these proteins as well [166].

Importantly, most enzymatically catalyzed PTMs have intimate connections to protein intrinsic disorder, as PTM sites targeted by modifying enzymes are commonly placed within IDRs. This is illustrated by phosphorylation, for which bioinformatics analysis revealed that many protein phosphorylation sites were located in regions that were structurally characterized as IDRs [167,168]. Furthermore, there is a high correspondence between the prediction of disorder and the occurrence of phosphorylation [169], and amino acid compositions, sequence change, complexity, and hydrophobicity, as well as many other sequence features of the regions adjacent to phosphorylation sites are very similar to those of IDRs [169]. In addition to phosphorylation, several other PTM types, such as acetylation, fatty acid acylation, methylation, protease digestion, and ubiquitination, have also been observed to preferentially occur within IDRs [45,167,168,170]. These observations indicate that in eukaryotic cells, localization of sites targeted for various PTMs show a strong preference for IDRs, making these sites easily accessible to modifying enzymes and explaining the functional promiscuity of those enzymes, where a single enzyme could bind to and modify a wide variety of protein targets.

### 4.3. Intrinsic Disorder, Structural Heterogeneity, Multifunctionality, and Binding Promiscuity

Importantly, protein intrinsic disorder has multiple flavors, as proteins have different levels and depths of disorder, and different parts of a protein can be (dis)ordered to different degrees [42]. This heterogeneity of disorder can be summarized by rephrasing the famous opening line of Leo Tolstoy’s novel Anna Karenina: “All ordered proteins are alike; each disordered protein is disordered in its own way”. In fact, IDPs/IDRs can exist in the extended (coil- or pre-molten globule-like) or collapsed (molten globule-like) forms [2,5,12,13,15,27,171,172,173], and an IDP/IDR can be more or less compact and possess smaller or larger amounts of flexible secondary/tertiary structures [2,5,12,13,173,174]. Furthermore, a typical IDP/IDR is not structurally homogeneous and instead might contain a multitude of potentially foldable, partially foldable, differently foldable, or non-foldable structural elements [3,22], indicating that foldability (or structure-coding potential) is non-homogeneously distributed within the amino acid sequences of a protein. One should also keep in mind that this distribution of differently (dis)ordered regions is constantly changing in time, and a given segment of a protein molecule can potentially show different structures or lack of structure at different time points [3,22].

Therefore, protein structure represents a highly dynamic and very heterogeneous entity, where not only the entire protein molecule is expected to be disordered to different degrees, but various protein segments (even rather short ones) can be differently disordered as well [3,22,109,175,176,177]. Such a mosaic structural architecture of a protein molecule can be considered as a set of foldons (regions capable of spontaneous folding), non-foldons (segments that do not fold), semi-foldons (regions that are always in a semi-folded state), inducible foldons (segments that can gain structure (at least partially fold) upon interaction with binding partners), inducible morphing foldons (regions capable of folding to the different structures upon interaction with different binding partners), and unfoldons (important but less stable parts of ordered proteins that must unfold (or undergo order–disorder transition, at least partially) in order to make the protein active) [3,22,109,175,176,177]. The distribution of these variously (dis)ordered segments (foldons, non-foldons, inducible foldons, inducible morphing foldons, semi-foldons, and unfoldons) is constantly changing in time, and the entire protein has a highly dynamic and morphing structure that is not rigid or crystal-like [3,22,109,176,177]. Furthermore, many proteins exist as complex structural hybrids possessing ordered and differently disordered domains, thereby defining another level of structural heterogeneity crucial for their functions [16]. Therefore, it is clear that the classification of proteins as ordered and disordered is an obvious oversimplification, as the structure-disorder space of a protein represents a continuum, with no obvious boundary between order and disorder [3,176].

It is clear that such complex, highly dynamic, mosaic-like structural organization of proteins is also reflected in complex disorder-based functionality of proteins, as all the differently (dis)ordered structural segments of proteins (foldons, non-foldons, inducible foldons, inducible morphing foldons, semi-foldons, and unfoldons) might have very different functions. Furthermore, since all these foldons, semi-foldons, non-foldons, inducible foldons, inducible morphing foldons, and unfoldons can be found within one protein molecule, one can clearly see that a protein with such a heterogeneous structure is inherently multifunctional. Therefore, the aforementioned protein structural continuum defines protein multifunctionality. These considerations constitute the basis for a “protein structure-function continuum” model, where a functional protein exists as a dynamic conformational ensemble characterized by a broad spectrum of structural features possessing different functionalities, and provides a global link between the protein structure and function [178].

Among the important functional features of IDPs/IDRs residing on their lack of stable structure are their ability to serve as hub proteins, i.e., nodes in protein–protein interaction networks that have a very large number of connections to other nodes [179,180,181,182,183,184,185], to bind partners with both high specificity and low affinity [186], to be engaged in promiscuous interactions with unrelated partners such as other proteins, small molecules, and nucleic acids [187], to contain molecular recognition features (MoRFs), which are short binding regions located within longer disordered regions that can fold upon interaction with a partner [179,188,189,190], to adopt different structures upon binding to different partners [10,187,191,192,193,194,195], to form fuzzy complexes, where a significant part of an IDP continues to be disordered even in the bound state outside the binding interface [158,196,197,198,199,200,201], to act as dynamic and sensitive “on-off” switches [198], and to be able to return to their highly dynamic and pliable conformations after the completion of a particular function [3,22].

Disorder-based interactions are commonly combinatorial and promiscuous in nature, and such combinatorial and promiscuous interactivity defines the multifunctionality of IDPs/IDRs. An illustrative example of this concept is given by the GPCR–G-protein signaling system, which in humans includes more than 800 G-protein-coupled receptors (GPCRs) [202,203,204,205] and a large set of intracellularly located guanine nucleotide-binding proteins (G-proteins), which are heterotrimers composed of α, β, and γ subunits, with their Gα subunit being diversified even further, as there are four major families (Gαs, Gαi, Gαq, and Gα12) encoded by 16 human genes [204,206,207]. Furthermore, the complexity of this system goes far beyond a multitude of pairwise ligand–GPCR and GPCR–G-protein interactions, as one GPCR can recognize more than one extracellular signal and interact with more than one G-protein and one ligand can activate more than one GPCR, and multiple GPCRs can couple to the same G-protein [208]. The biological importance of this system cannot be overemphasized, as it recognizes a multitude of extracellular ligands, triggers a variety of intracellular signaling cascades in cellular responses to hormone neurotransmitters, ions, photons, and other environmental stimuli, and is responsible for vision, olfaction, and taste. In fact, more than 1000 natural and artificial extracellular ligands, ranging from photons to amines, lipids, nucleotides, organic odorants, peptides, and proteins can interact with and activate GPCRs [205,206], and these signals are used to initiate a wide spectrum of intracellular signaling cascades via interaction of an activated GPCR with a Gα subunit, which is a member of one of the four major Gα families. This results in the activation or modulation of various downstream effector proteins and key secondary messengers [206,209,210]. The combinatorial and promiscuous nature of this system is further reflected in the fact that interactions between the activated GPCRs and Gα proteins are characterized by complex coupling selectivity, where several different GPCRs can pair with the same Gα protein and one GPCR can combine with more than one Gα protein. All these features define the GPCR–G-protein system as a cellular “control panel” capable of detecting an exceptionally diversified set of molecules outside the cell and initiating a broad variety of intracellular signaling cascades in response [211]. This combinatorial promiscuity is further amplified and, in fact, is explained by the presence of intrinsic disorder and associated with high conformational flexibility of the members of this system. In fact, it was shown that the cytoplasmic and extracellular regions of GPCRs encompass numerous IDRs, multiple disorder-based binding sites, and abundant PTM sites, and typically have multiple isoforms generated by alternative splicing [208,212]. Similarly, all human G-proteins contain noticeable levels of functional intrinsic disorder, include numerous sites of various PTMs, include disorder-based interaction sites, and exist as multiple isoforms generated by alternative splicing [208]. Furthermore, both GPCRs and G-proteins often undergo function-associated conformational changes that range from domain motion to binding-induced disorder-to-order transitions. In other words, the multifunctionality of these major players of the GPCR–G-protein system is determined by the fact that all these proteins exist as numerous and highly dynamic conformational/basic, inducible/modified, and functioning proteoforms [208].

It is important to note that combinatorial promiscuity can not only be used to describe the assembly of operating protein systems, but also to define the outputs of action of the corresponding promiscuous reconfigurable signaling networks at the organismal level. This point is illustrated by the action of a family of important chemosensory GPCRs, the olfactory receptors (ORs), which are located in the nasal olfactory epithelium and are responsible for the sense of smell. In humans, ~400 ORs are used to discriminate at least one trillion olfactory stimuli [213]. Obviously, such a situation is incompatible with the scenario, where each dedicated OR recognizes one specific odorant molecule. Instead, ORs of a particular type can display broad sensitivities to different odorants (i.e., it can recognize multiple odorants), each odorant can promiscuously bind to receptors of many types (i.e., one odorant is recognized by multiple ORs), and different odorants are recognized by different combinations of ORs [214,215]. Therefore, odorants are discriminated in a combinatorial manner [214], where ORs bind odorants promiscuously with different affinities, and the corresponding combinatorial rules define the output signal sent to the brain.

## 5. Protein Intrinsic Disorder and Evolution of Multicellularity

### 5.1. Intrinsic Disorder and Proteoforms

It is very likely that IDPs played important roles in various stages of the origin of life and evolution, being involved in prebiotic evolution preceding the origin of Tibor Ganti’s Chemoton, a suspected precursor to the first universal common ancestor and, subsequently, to later stages of evolution, including the early origin of complex multicellularity and the ensuing bilateria during the Cambrian explosion ~571 million years ago [216,217,218,219]. The cornerstone of modern evolutionary theory is the existence of a last universal common ancestor (LUCA), which is a hypothetical common ancestral cell from which the three domains of life—the Bacteria, the Archaea, and the Eukarya—have originated [78,79] and which lived roughly 3.5 billion years ago, as it follows from a comprehensive computational analysis using model selection theory without making an assumption that sequence similarity indicates a genealogical relationship [79]. The existence of LUCA is supported by multiple observations [79,220,221,222], such as the following:

- The agreement between phylogeny and biogeography;
- The correspondence between phylogeny and the paleontological record;
- The existence of numerous predicted transitional fossils;
- The hierarchical classification of morphological characteristics;
- The marked similarities between biological structures with different functions (that is, homologies); and
- The congruence of morphological and molecular phylogenies.

Complex multicellularity implies the presence in the organism of multiple differently specialized cells responsible for the formation of tissues and organs. Among the molecular mechanisms required for the development of complex multicellularity are the means to increasing the size of the functional proteome relative to the encoding genome that encodes it, which also represents an important phenomenon behind the observation that the complexities of biological systems are mostly determined by their proteome sizes and not by the dimensions of their genomes [223]. This can be illustrated by the gene–protein relationship in Homo sapiens [224,225,226,227,228], where the number of protein-coding genes ranges between 20,000 and 25,000 [132], but the actual number of functionally different proteins is in the range of a few million [229] to several billion [230]. The required structural and functional diversification of a proteome can be achieved by allelic variations (i.e., single or multiple point mutations (amino acid polymorphisms), indels, single nucleotide polymorphisms (SNPs)), alternative splicing, mRNA editing, and other pre-translational mechanisms affecting mRNAs, as well as by a wide spectrum of various PTMs of a polypeptide chain. As a result, a single gene encodes a set of distinct protein molecules, known as proteoforms [230]. Since all these aforementioned mechanisms are associated with some changes in the physicochemical structure of a polypeptide chain, the resulting proteoforms have induced or modified natures. Importantly, protein structural diversity is further enhanced by intrinsic disorder and functionality, giving rise to the conformational or basic proteoforms and functioning proteoforms, respectively [231]. However, since many PTM sites are preferentially located within the IDRs [169,232], since mRNA regions affected by alternative splicing predominantly encode IDRs [158], since IDPs/IDRs act as highly promiscuous binders [5,11,12,13,14,15,22,24,167,168,174,179,188,198,233,234,235,236,237,238,239], and since IDPs/IDRs are characterized by exceptional spatiotemporal heterogeneity, proteins and protein regions without unique structures represent a very rich source of proteoforms [231].

### 5.2. Casual Emergence

Since multicellular organisms represent complex systems, their organization and behavior are driven by casual emergence, where the higher scale of a system has stronger causal relationships than its underlying lower scales, allowing macroscales to reduce noise in causal relationships, thereby leading to stronger causes at the higher scale level [240]. Emergence is defined as the appearance of a multi-part, complex system, the behavior of which cannot be derived, predicted, or understood by looking at the behavior of its parts. It is one of the characteristic features of complex systems, the behavior of which is determined by a set of common rules [241]:

Complex systems contain many heterogeneous components involved in nonlinear interactions, where a small perturbation may cause a large effect, a proportional effect, or even no effect at all. Therefore, the behavior of a complex system cannot be expressed as the sum of the behaviors of its parts (or of their multiples):

- The constituents of a complex system are interdependent;
- A complex system possesses a structure spanning several scales and may be nested, i.e., the components of a complex system may themselves be complex systems;
- A complex system is capable of emergent behavior, which is unanticipated behavior shown by the system, for example, the arising of novel and coherent structures, patterns, and properties during the process of self-organization;
- Complexity involves an interplay between chaos (disorder) and order;
- Complexity involves an interplay between cooperation and competition, and complex systems contain both positive (amplifying) and negative (damping) feedback;
- Complex systems may have a memory. In other words, the history of a complex system may be important, since due to their dynamic nature, complex systems change over time, and prior states may have an influence on present states (for example, no two genetically identical mice or even two single cells that share the exact same DNA sequence are absolutely identical because of environmental influences, random variations in gene expression, and epigenetic modifications).

It was emphasized that IDPs/IDRs are complex “edge of chaos” systems, as their behavior obeys the aforementioned regulations. “Heterogeneous nature of IDPs is obvious. In fact, IDPs and IDRs are heterogeneous at multiple levels. Globally, they can be compact or extended and their major structural components are heterogeneous too, giving rise to foldons, induced foldons, semi-foldons and non-foldons. These structural components can be independent or interdependent, and they are able to interact nonlinearly. Functional misfolding represents an illustration of the interplay between cooperation and competition. The spatiotemporal complexity of IDPs/IDPRs is further increased by the fact that they and their structural components are always moving between order and disorder. IDPs are able to sense various stimuli and response to these stimuli via corresponding structural changes, where even smallest environmental perturbations might produce large structural and functional outcomes. IDPs/IDPRs possess emergent behavior, since under some conditions they are able to undergo self-organization via stimuli-induced disorder-to-order transitions. Finally, MoRFs, SLiMs and PreSMos represents a memory of the IDP, since they are transiently populated in the non-bound state and may have a profound influence on IDP binding mechanism and on the resulting bound state. All this supports the hypothesis that IDPs/IDPs are positioned at the edge of chaos” [22].

Since in the case of casual emergence, groups of features influence the future of a system together, rather than separately, this mechanism is crucial for governing reliable large-scale responses, such as determining the fate of a single cell, defining intercellular communication and collaboration to form tissues and organs, and even delineating the behavior reaction of an organism in responses to external stimuli. Although casual emergences were shown to be present in protein–protein interaction (PPI) networks (interactomes) of both prokaryotes and eukaryotes (where a cluster of PPIs can be replaced by a single “macro-node” capable of conducting the same job as the collective), it was more evident in eukaryotes and especially in the complex multicellular eukaryotic organisms [242]. These findings indicated that the more complex organisms tend to more often use higher organization levels of their networks for casual roles, thereby becoming more tolerant to noise and indeterminism of their microscales, as macroscales of interactomes are more resilient than microscales [242]. In this way, their noisy microscales do not serve as primary determinants of the phenotypic outcomes ranging from body structure and body shape to behavior [242]. Importantly, this increase in the casual emergence in complex eukaryotes can explain a rather counter-intuitive observation that the effectiveness of the protein interactomes measured as the effective information that serves as an information–theoretic network quantity based on the entropy of random walker behavior on a network and is reflected in the certainty (or uncertainty) contained in connectivity of analyzed network [243] decreased in moving from prokaryotes to eukaryotes [242]. It is very likely that the observed increase in casual emergence in complex eukaryotes is linked to the higher levels of intrinsic disorder in their proteomes. In fact, although due to the abundant presence of IDPs/IDRs, eukaryotic interactomes at their microscales become noisy, more stochastic, and less effective over evolutionary time [242], the formation of macro-nodes that define the macroscale structure of the corresponding interactomes is likely to be driven by protein intrinsic disorder.

**Figure 7:**
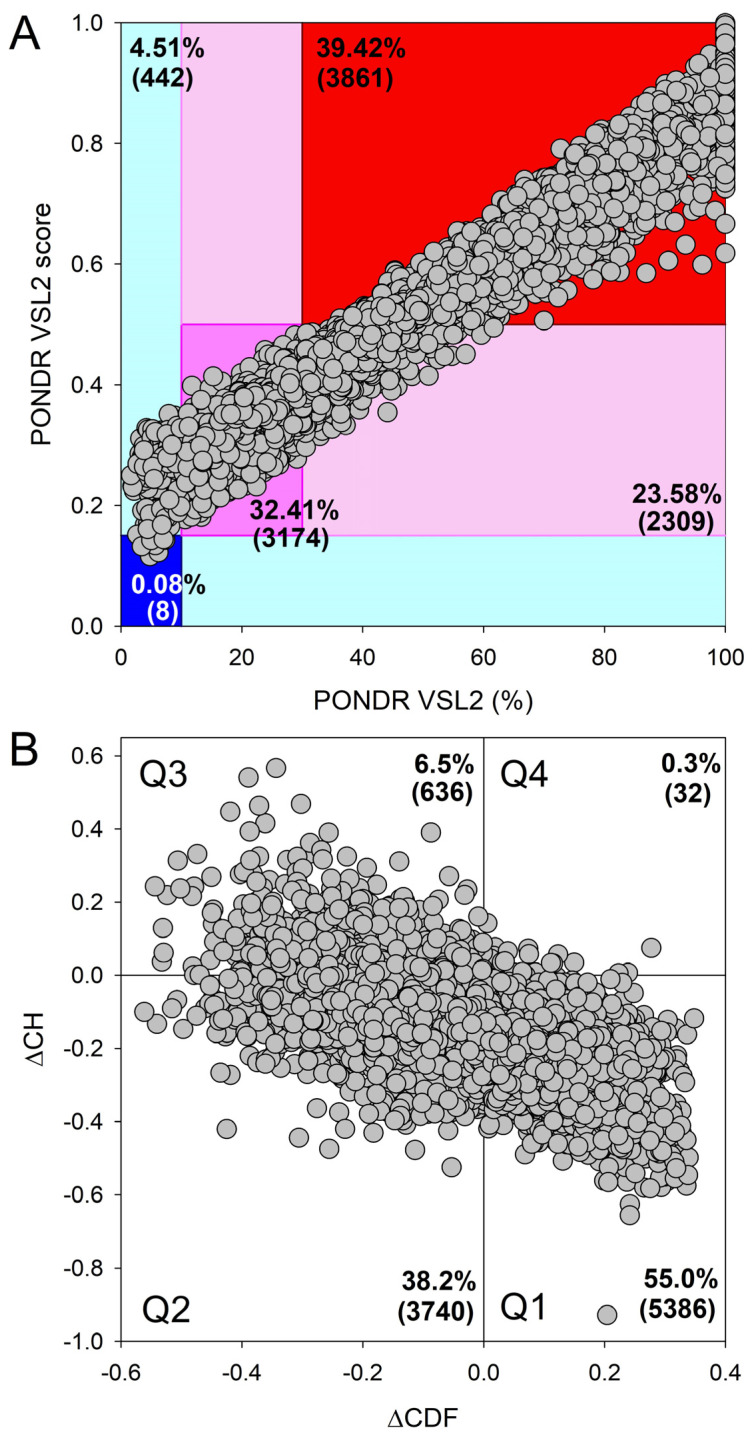
Multifactorial intrinsic disorder analysis of the entire proteome of amoeboid holozoan *Capsaspora owczarzaki* containing 9794 proteins. (**A**) PONDR^®^ VSL2 Score vs. VSL2 PONDR^®^ (%) analysis. PONDR^®^ VSL2 (%) is a percent of predicted intrinsically disordered residues (PPIDRs), i.e., residues with disorder scores above 0.5. PONDR^®^ VSL2 score is the average disorder score (ADS) for a protein. Based on these parameters, query proteins are classified as ordered (PPIDR < 10%; ADS < 0.15), moderately disordered (10% ≤ PPIDR < 30%; 0.15 ≤ ADS < 0.5), and highly disordered (PPIDR ≥ 30%; ADS ≥ 0.5). Color blocks indicate regions in which proteins are mostly ordered (blue and light blue), moderately disordered (pink and light pink), or mostly disordered (red). If the two parameters agree, the corresponding part of the background is dark (blue or pink), whereas light blue and light pink reflect areas in which the predictors disagree with each other. The boundaries of the colored regions represent arbitrary and accepted cutoffs for ADS (y-axis) and the percentage of predicted disordered residues (PPIDR; x-axis). For comparison, in the human proteome, 0.4%, 5.1%, 33.7%, 21.0%, and 40.1% of proteins are located within blue, light blue, pink, light pink, and red segments, respectively. This distribution observed in the human proteome is remarkably close to the distribution reported here for the *C. owczarzaki* proteins. (**B**) Charge-Hydropathy and Cumulative Distribution Function (CH-CDF) analysis of *C. owczarzaki* proteins. The CH-CDF plot is a two-dimensional representation that integrates both the CH plot, which correlates a protein’s net charge and hydrophobicity with its structural order, and the CDF, which cumulates disorder predictions from the N-terminus to the C-terminus of a protein, offering insight into the distribution of disorder residues. The y-axis (ΔCH) represents the protein’s distance from the CH boundary, indicating the balance between charge and hydrophobicity, while the x-axis (ΔCDF) represents the deviation of a protein’s disorder frequency from the CDF boundary. Proteins are then stratified into four quadrants: Quadrant 1 (bottom right) indicates proteins likely to be structured; Quadrant 2 (bottom left) includes proteins that may be in a molten globule state or lack a unique 3D structure; Quadrant 3 (top left) consists of proteins predicted to be highly disordered; Quadrant 4 (top right) captures proteins that present a mixed prediction of being disordered according to CH but ordered according to CDF. For comparison, 59.1%, 25.5%, 12.3%, and 3.1% of human proteins are located within quadrants Q1, Q2, Q3, and Q4, respectively. This indicates that although the *C. owczarzaki* and human proteomes contain comparable fractions of ordered proteins, there are noticeably more native molten globules and noticeably less highly disordered proteins in the *C. owczarzaki* proteome.

In an attempt to understand what might trigger the transition to multicellularity, the genome and proteome of a single cellular eukaryote, the amoeboid holozoan *Capsaspora owczarzaki*, which is one of the evolutionarily closest relatives of the first multicellular animals, were investigated [244]. The researchers paid special attention to the genes/proteins involved in the transcriptional regulation, as untangling the early evolution of transcription factors (TFs) is critical for understanding the origin of metazoans and animal development [244]. This analysis revealed that *C. owczarzaki* contains more transcription factors than any other known single-cellular organism and that the transcription factors found in this organism are already organized in specific networks that are often found in multicellular animals as well. It was also emphasized that the complexity of the repertoire of transcription factors in *C. owczarzaki* “is strikingly high, pushing back further the origin of some transcription factors formerly thought to be metazoan specific” [244]. Therefore, it seems that at least some means (in the form of the specific TF-containing networks) required for animal development were present even before the appearance of multicellularity, suggesting that the switch to multicellularity was driven by devising new ways of gene regulation rather than by the appearance of more new genes [244]. Figure 7 illustrates the remarkably high level of global intrinsic disorder content in the *C. owczarzaki* proteome, which is comparable to that of the human proteome.

Phenotypic changes in animal lineages are linked to the gain, loss, and modification of gene regulatory elements [245]. Often, such regulation is achieved using cis-regulatory conserved non-exonic elements (CNEEs), which are evolutionarily conserved yet do not overlap with any, coding or noncoding, mature transcript [245], and which show a strong linkage with trait/disease-associated single nucleotide polymorphisms [246]. By analyzing genome-wide sets of putative regulatory regions in five vertebrates, including humans, to infer the branch on which each CNEE came under selective constraint, it was shown that the evolution of gene regulatory elements is characterized by the presence of three extended periods [245]. It was indicated that instead of the gradual changes in the frequencies of regulatory elements over the past 650 million years, the evolution of CNEEs saw three different eras, with early vertebrate evolution lasting from the vertebrate ancestor until about 300 million years ago (when mammals split with birds and reptiles), characterized by regulatory gains near transcription factors and developmental genes. The second period, which was between 300 and 100 million years ago, was characterized by the replacement of the first trend by a high frequency of regulatory innovations near extra-cellular signaling genes, and then, from 100 million years ago, the third period, which affected placental mammals, was characterized by an increase in regulatory innovations for genes involved in post-translational protein modification [245]. Although CNEEs, by default, are non-coding elements, peculiarities in their evolution indicate the crucial roles of regulatory gains of genes mostly encoding proteins with high levels of intrinsic disorder, such as transcription factors and receptors, or proteins mostly acting by modifying functionality of IDPs/IDRs, such as proteins related to PTM control. Therefore, this specific CNEE evolution emphasizes the importance of IDPs/IDRs in animal evolution.

### 5.3. Intrinsic Disorder, Noise/Stochasticity of Transcriptional Regulation, and Development

**Figure 8:**
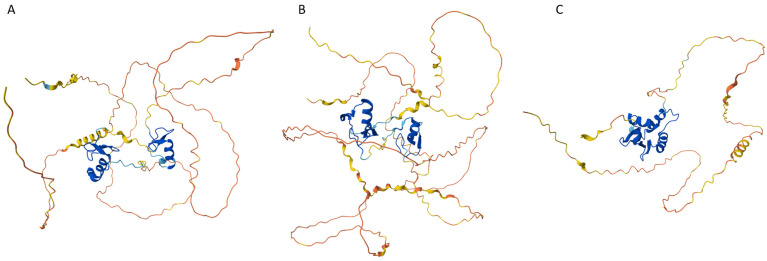
Three-dimensional structural model generated by AlphaFold [156] for mouse GATA1 (UniProt ID: P17679) (**A**), GATA2 (UniProt ID: O09100) (**B**), and PU.1 (UniProt ID: P17679) (**C**) proteins. Structures are colored according to the model confidence, with blue, cyan, yellow, and orange colors corresponding to the regions with very high, high, low, and very low confidence, respectively.

The examples in the preceding section illustrate the overall complexity of the disorder-based organizing principles of biological networks, which are inherently noisy, and, being promiscuous, rather indiscriminative, and insensitive to the fine details, use combinatorial and fuzzy logics to solve various cellular and organismal queues. Furthermore, all these observations hint at the idea that biological actions are stochastic/noisy, and part of this stochasticity/noisiness is determined by the presence of intrinsic disorder in acting proteins. Importantly, this biological noisiness represents an important driving factor for development and evolution. This concept can be illustrated by considering the dynamical landscape defining stochastic determination of cell fate during, for example, the differentiation of mouse hematopoietic stem cells into specialized blood cell types via the formation of multipotent progenitor cells first. One of these multipotent progenitor cells, the myeloid progenitor cell, can differentiate either into erythrocytes or precursors of certain white blood cells, with the choice between erythroid and myelomonocytic fates being determined by the interplay between the two lineage-determining transcription factors, GATA1 and PU.1 [247]. In this bifurcation, multipotent progenitor cells expressing more GATA1 will end up in the erythroid state, whereas the myeloid state is triggered by higher levels of PU.1 expression. The complexity of this relatively simple system regulation is determined by the fact that it has sensitive feedback, as GATA1 and PU.1, being self-promoting, can inhibit each other’s expression. The dynamics of the resulting binary fate decision system represent an illustration of the phenomenon of “multilineage priming”, where a gene-circuit generates stable attractors corresponding to the erythroid and myelomonocytic fates, as well as an uncommitted metastable state characterized by co-expression of both TFs [247]. Here, commitment to a particular cell fate occurs in two stages, where at the first stage, the progenitor state is destabilized in an almost symmetrical bifurcation event, resulting in a poised state at the boundary between the two lineage-specific attractors; second, the cell is driven to the respective, now accessible attractors” [247]. It was also shown that another TF, GATA2, which is antagonistic to PU.1 but boosts GATA1 expression, plays an important role in the differentiation of mouse hematopoietic stem cells by adding to the transcription noise [248]. Here, infrequent stochastic bursts of transcription lead to the co-expression of these antagonistic TFs in the majority of hematopoietic stem and progenitor cells, thereby opening a possibility for the cells to reach both target lineages more reliably instead of being stuck on one or another track [248]. In other words, the noisiness of the transcription regulation represents an important way of keeping all the cell-fate options open, where a system maintains a temporally stable probability of cells in every available transcriptional state [248]. Since the major players in this system are transcription factors, it is not surprising that GATA1, GATA2, and PU.1 are highly disordered, as illustrated by Figure 8. It is tempting to assume that this system serves as an illustration of the utilization of protein intrinsic disorder in noisy transcriptional regulation required for cell differentiation.

## 6. Conclusions

This article analyzes some of the potential implementations of intrinsic disorder in the origin of life and evolution. Clearly, the views presented here are rather personalized and admittedly subjective. With a very high probability, some aspects are incompletely covered, and other aspects related to this subject are missed. However, one message is absolutely clear: neither the origin of life nor evolution would be possible without protein intrinsic disorder. In fact, IDPs, with their highly heterogeneous structural organization, related multifunctionality, and enormous interactivity, seem to be perfect life organizers and evolution drivers. Even in a perfect world of highly ordered biological catalysts (enzymes), intrinsic disorder cannot be ridiculed, since primordial IDPs were the entities that started the molecular evolution of modern enzymes. In fact, the chances that a perfect catalyst with a unique 3D structure responsible for a unique catalytic function would spontaneously appear on the primordial Earth are negligible. Instead, one can easily imagine a scenario where an extremely floppy polypeptide capable of lousy substrate recognition could have very sloppy catalytic activity. If the rate of the resulting floppy–sloppy “pseudo catalytic” reaction would be even slightly higher than the rate of the corresponding spontaneous, non-catalyzed reaction, one would have an excellent starting point for evolutionary improvement. Obviously, not everything would evolve into highly ordered specialized machines, and numerous modern biological processes are critically dependent on the floppo-sloppiness of IDPs. Life is not something frozen in time and space, and biological processes (especially those in more complex organisms) are not controlled by the precise “chain of command”, being instead stochastic in nature. Acting as crucial constituents of terrestrial life, IDPs are “edge of the chaos” systems capable of emerging behavior. IDP-driven, IDP-governed, or at least IDP-related emergence is everywhere and has multiple forms and levels. The apparent re-introduction of intrinsic disorder associated with the evolutionary emergence of complex eukaryotic organisms might represent a natural way of addressing the second law of thermodynamics, where the emergence of such organismal complexity is compensated by a noticeable leap in the protein disorder content that provides a necessary increase in the system’s entropy. Evolution is rooted in intrinsic disorder, as IDPs were crucial for the origin of life and the emergence of protocells, drove the split between prokaryotes and eukaryotes, and orchestrated the emergence of multicellularity.

## Data Availability

Not applicable.
